# Presence and dynamics of leptin, GLP-1, and PYY in human breast milk at early postpartum

**DOI:** 10.1002/oby.20345

**Published:** 2013-05-29

**Authors:** Jessica Schueler, Brenda Alexander, Ann Marie Hart, Kathleen Austin, D Enette Larson-Meyer

**Affiliations:** 1 Department of Family and Consumer Sciences, University of WyomingLaramie, Wyoming, USA; 2 Department of Animal Science, University of WyomingLaramie, Wyoming, USA; 3 Faye Whitney School of Nursing, University of WyomingLaramie, Wyoming, USA

## Abstract

**Objective:** The presence of appetite hormones, namely glucagon-like peptide-1 (GLP-1), peptide YY (PYY), and leptin in breast milk may be important in infant feeding regulation and infant growth. This study evaluated whether concentrations of GLP-1, PYY, and leptin change across a single feeding (from fore- to hindmilk), and are associated with maternal and infant anthropometrics.

**Design and Methods:** Thirteen postpartum women (mean ± SD: 25.6 ± 4.5 years, 72.0 ± 11.9 kg) provided fore- and hindmilk samples 4-5 weeks after delivery and underwent measurements of body weight and composition by Dual X-ray Absorptiometry. GLP-1, PYY, and leptin concentrations were measured using radioimmunoassay, and milk fat content was determined by creamatocrit.

**Results:** Concentration of GLP-1 and content of milk fat was higher in hindmilk than foremilk (*P* ≤ 0.05). PYY and leptin concentrations did not change between fore- and hindmilk. Both leptin concentration and milk fat content were correlated with indices of maternal adiposity, including body mass index (*r* = 0.65-0.85, *P* < 0.02), and fat mass (*r* = 0.65-0.84, *P* < 0.02). Hindmilk GLP-1 was correlated with infant weight gain from birth to 6 months (*r* = −0.67, *P* = 0.034).

**Conclusion:** The presence of appetite hormones in breast milk may be important in infant appetite and growth regulation.

## Introduction

In addition to providing optimal nutrition for the infant, breastfeeding offers numerous health benefits that are unmatched by formula. Breastfeeding not only decreases the risk for ear infections, sudden infant death syndrome, lower respiratory tract infections, diarrhea, and vomiting, but also reduces risk for obesity later in life 1–2. The obesity-protective effect of breastfeeding may be because of components found in breast milk or differences in feeding practices (breastfeeding vs formula-feeding from a bottle). Breastfed infants, for example, show evidence of appetite regulation not observed in formula-fed infants 3–4. They consume fewer calories than formula-fed babies 5, appear to adjust to the energy content of milk 6, and have lower weight-for-length ratios, on average, at 7-24 months of age than formula-fed infants 6. Evidence of an effect on weight regulation extends into early adolescence. A recent sibling study found that 14-year-olds who were breastfed were, on average, 13 pounds lighter than their formula-fed siblings 7.

The discovery that two key appetite-regulating hormones, leptin and ghrelin, are present in breast milk—in addition to a variety of bioactive molecules including immunoglobulins, growth factors, and enzymes—provides an attractive explanation for the ability of breast milk to regulate infant body weight. Bouret 8 has recently suggested that the presence (or lack) of appetite hormones may permanently affect the appetite-regulating system of the infant by affecting the development of appetite-regulating centers in the brain, specifically the hypothalamus. These differences in appetite hormone exposure may create permanent changes in the way the brain reacts to appetite hormones and satiety cues 8.

Leptin, a 146 amino acid peptide hormone produced in white adipose tissue and stomach 9, has been detected in breast milk at concentrations of 0.2-8.0 ng/mL 10,11. In postpartum mothers, circulating leptin correlates with body adiposity 13 and is believed to serve as an anorexigenic signal when adipose stores are high. Additionally, the concentration of leptin in breast milk is correlated to maternal BMI 14. A second anorexigenic hormone, peptide YY (PYY), was detected in breast milk by both radioimmunoassay (RIA) and high-pressure liquid chromatography at a concentration of 28 ng/mL in the early 1990s 15. A similar anorexigenic hormone, glucagon-like peptide-1 (GLP-1), is yet to be detected in milk. GLP-1 and PYY are much smaller peptides of 31 and 36 amino acids, respectively, and both are known to be produced in the mucosal L-cells of the intestine 16–17. Both hormones are released in response to the presence of nutrients in the intestine 16–17 and, once released, act on appetite centers in the brain to inhibit food intake at subsequent meals 18–19.

A hallmark of breast milk is that its composition varies widely and is not static over time 20. It is well known that the fat content of foremilk is lower than hindmilk 21. Limited research suggests that the concentrations of PYY 15 and leptin 12 in human milk decrease during the first year after birth. Additionally, a recent study suggests that the appetite-stimulating hormone, ghrelin, decreases in concentration over the course of a single feeding 11. The changes in fat and hormone contents of milk suggest these bioactive elements may play a physiological role in appetite regulation.

The purpose of this pilot study was to determine whether GLP-1 is present in human breast milk and whether the appetite-suppressing hormones, GLP-1, PYY, and leptin, change in concentration from fore- to hindmilk. We hypothesized that breast milk concentrations of GLP-1 and PYY would increase with duration of feeding, sending a signal of increased satiety to the infant. However, as leptin is a long-term regulator of appetite, we hypothesized that concentrations would remain stable from fore- to hindmilk. A second purpose of the study was to examine associations between the concentrations of appetite-suppressing peptides in milk and both maternal adiposity and infant growth. We hypothesized that plasma hormone concentrations influenced by maternal adiposity would be similarly affected in milk. Lastly, if appetite hormones in breast milk play a role in the regulation of infant energy intake, we hypothesized that milk GLP-1, PYY, and leptin would negatively correlate with infant weight gain.

## Methods and Procedures

Thirteen healthy postpartum mothers and their infants participated in this study from fall 2009 to spring 2011. To qualify for the study, participants had to be first-time mothers exclusively breastfeeding, at least 18 years of age with normal thyroid function (thyroid stimulating hormone [TSH] concentration between 0.47-4.68 μIU/mL) and hemoglobin concentration between 12.0-16.0 g/dL. Women were not eligible if they had given birth to multiples, had major pregnancy complications (including gestational diabetes, preeclampsia, and/or severe bleeding), reported having any major diseases, had resting blood pressure above 140/90 mm Hg, were taking any medication or herbal supplement that affect metabolism, or had any signs of psychological disorders or substance abuse. Mothers who smoked or had smoked during the previous year were also excluded. The study was approved by the Institutional Review Board of the University of Wyoming. Volunteers were fully informed of possible risks of all procedures before providing written informed consent.

### Overview of testing

Postpartum women visited the laboratory between 29-38 days after delivery to provide fore- and hindmilk samples. Anthropometric measurements of both mothers and infants were collected. Specifically, maternal body weight was obtained in minimal clothing prior to milk collection using a digital scale (Tanita, Tokyo, Japan). Height was measured in bare feet with a stadiometer (Invicta Plastics, Leicester, England). Infant body mass was measured using an UltraScale MBSC-55 (My Weigh USA, Phoenix, Arizona, USA) when clothed in only a dry diaper. Infant length was measured as the parallel distance from the head to the heels as marked on paper. Maternal body composition was measured using Dual X-ray absorptiometry (DXA) (Lunar Prodigy, GE Healthcare, Fairfield, Connecticut, USA). Self-reported infant birth weight/length, pre-pregnancy weight, and weight gain during pregnancy were also collected.

Infants were also followed longitudinally for one year after birth. Anthropometric measurements, as previously described, were obtained at 6 and 12 months after delivery. A parent-reported infant weight from a physician visit was accepted in place of weighing for infants whose families were no longer living in the local area or were unable to complete the 6 or 12 month follow-up at the scheduled time. Infant weight gain was calculated by subtracting reported birth weight from the infant weight measured at the milk collection and follow-up visits. Infant weights were calculated as percentiles for weight-for-age and weight-for-length using the sex-based World Health Organization growth charts (WHO Anthro 3.2.2; WHO, Geneva, Switzerland).

### Collection of milk samples

Breast milk samples were collected in the morning between 7 and 10 AM, during the mother's usual nursing time. Mothers reported to the laboratory after an overnight fast of at least 10 hours. Milk was collected using a mechanical pump provided by the laboratory (miPump Single, The First Years, Dyersville, Iowa, USA) or the mother's own pump. Foremilk (at least 30 mL) was collected before feeding the infant and hindmilk (at least 30 mL) was collected from the same breast after feeding the infant for 6-10 minutes, depending on the mother's perceived milk availability and the infant's feeding rate. Immediately after collection, milk samples were placed on ice and the protease inhibitors aprotinin (37.5 μL/mL) (Millipore, St. Charles, Missouri, USA) and dipeptidyl peptidase-4 inhibitor (10 μL/mL) (Millipore) were added to milk samples for analysis of GLP-1 and PYY. Protease inhibitors were not added to samples used for analysis of leptin. Fat content of the milk samples was determined by creamatocrit analysis of whole fore- and hindmilk samples using an Autocrit Ultra 3 (Becton, Dickinson, and Company, Franklin Lakes, New Jersey, USA), according to the procedures reported by Lucas et al. 22. All samples were immediately cold-centrifuged at 4°C at 3500 rpm for 10 minutes. The top layer of fat was removed by vacuum using a Pasteur pipette attached to a Buchner flask. The remaining skimmed milk was then partitioned into Eppendorf tubes and stored at −80°C until sample analysis.

### Hormone assays

Leptin was analyzed using a human leptin RIA (Millipore) according to the manufacturer's directions as previously reported 11–12. Breast milk GLP-1 was analyzed with an RIA kit for total GLP-1 (Millipore). PYY was assayed with a human total PYY RIA (Millipore). To determine any assay interference from milk proteins, we analyzed sample dilutions to determine parallelism with the assay standards for GLP-1 and PYY. All samples were assayed in duplicate in a single assay with intra-assay variations of 7.5%, 6.2%, and 9.4%, and for GLP-1, PYY, and leptin, respectively.

### Statistics

Statistical analysis was performed with PASW version 18 (IBM, Armonk, NY, USA). Descriptive data were compared using the Student's two-sample *t-*test. Hormone concentrations in foremilk and hindmilk were compared using the Student's one-sample *t*-test. In this test, the relative change in concentration was calculated as (Hind-Fore)/Fore and compared to a null value of 0. Hormone and fat contents of fore- and hindmilk were compared to maternal BMI, % body fat, body weight, and fat mass, along with infant weight gain from birth to 12 months using Pearson correlation coefficients. *P*-values of 0.05 or less were deemed significant. Data are presented as arithmetic means ± SD.

## Results

The descriptive characteristics of the mothers and infants are shown in Table1. All mothers were in good general health, with TSH concentrations averaging 1.6 ± 0.7 μIU/mL, and no signs of anemia (average hemoglobin concentration of 14.4 ± 1.3 g/dL). The BMI of the mothers ranged from 20.4 to 33.0 kg/m^2^ and averaged 26.0 ± 4.2 kg/m^2^. All infants were born to term with an average weight of 3.3 ± 0.5 kg. A creamocrit analysis was missed on fore- and hindmilk samples of one participant, therefore data regarding fat content of milk are reported on *n* = 12. Additionally, because of study attrition, follow-up infant anthropometrics were not obtained on three infants at the 6- and 12-month follow-up (*n* = 10 for all 6- and 12-month data). Anthropometric data from a physician visit were used for 7 of the 31 infant anthropometric measures.

**Table 1:** Descriptive characteristics of mothers and infants

	Mean	SD	Minimum	Maximum
Mothers				
Age (y)	25.6	4.5	19	33
Height (cm)	166.58	8.57	154.10	179.20
Weight (kg)	71.95	11.78	52.80	92.30
BMI (kg/m^2^)	25.98	4.23	20.35	32.95
Waist Circumference (cm)	95.11	10.28	77.00	114.00
Body fat (%)	39.90	7.64	26.50	53.00
TSH (μIU/mL)	1.57	0.65	0.63	2.71
Hemoglobin (g/dL)	14.41	1.26	12.20	16.10
Infants (38% female)				
Birth Weight (kg)	3.32	0.47	2.71	4.23
Weight at ∼1 month (kg) (*n* = 11)	4.27	0.44	3.65	4.86

BMI, body mass index; TSH, thyroid stimulating hormone.

### Detection of hormones in breast milk

Detectable concentrations of GLP-1 and PYY were found in all milk samples (Table2). Leptin was detected in samples from all participants but one (Table2). For that participant, leptin was undetectable in both fore- and hindmilk samples. The average concentrations of GLP-1 and PYY in all milk samples were 14.1 ± 3.3 pM with a range of 6.3-21.9 pM and 39.2 ± 8.6 pg/mL with a range of 21.8-60.0 pg/mL, respectively. The average concentration of leptin in all milk samples was 1.0 ± 0.7 ng/mL with a range of 0.2-2.6 ng/mL, excluding one participant who had undetectable concentrations of milk leptin. This average did not change significantly (0.9 ± 0.7 ng/mL) when the lowest detectable concentration of leptin (0.1 ng/mL) was used for the participant with undetectable concentrations. Thus, the value of 0.1 ng/mL was used for concentrations of both fore- and hindmilk for this participant in all subsequent correlative analyses. Fat content of all milk samples averaged 13.6% with a range of 0.5-60.0% (Table2).

**Table 2:** Concentrations of hormones and fat content in fore- and hindmilk samples

	Foremilk	Hindmilk
PYY (pg/mL)	39.5 ± 8.4	38.9 ± 9.1
GLP-1 (pM)	12.7 ± 3.1*	15.4 ± 3.1
Leptin (ng/mL)	0.9 ± 0.7	1.0 ± 0.8
Fat (% by volume) (*n* = 12)	10.4 ± 13.2*	19.2 ± 17.2

GLP-1, glucagon-like peptide 1; PYY, polypeptide YY. Averages expressed as mean ± SD.

* Significant difference between foremilk and hindmilk (*P* ≤ 0.05).

### Changes in breast milk over the course of an individual feeding

**Figure 1:**
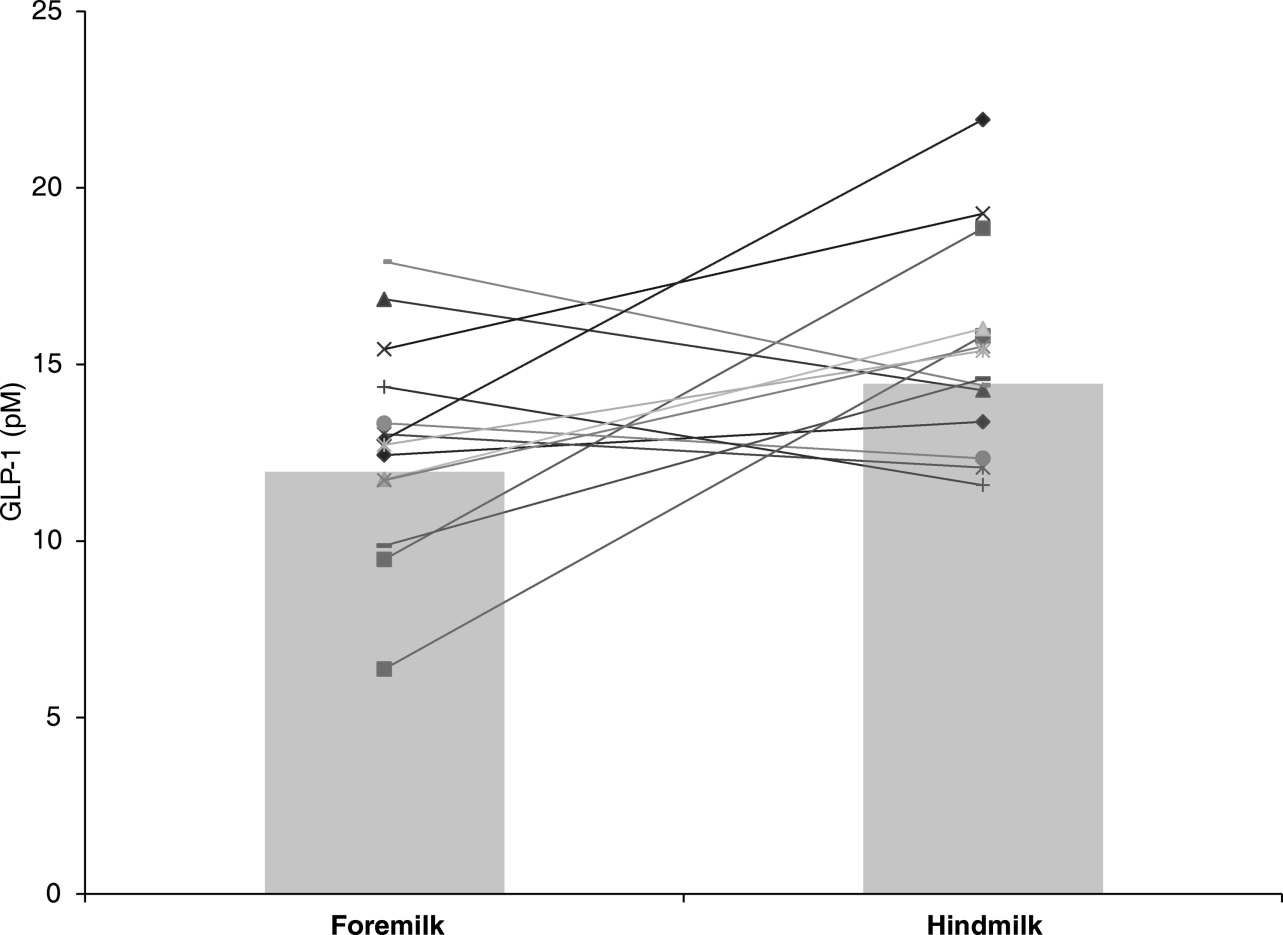
Concentration of GLP-1 in fore- and hindmilk as measured in 13 postpartum women 1 month after delivery. Each woman is represented by a line in the figure and bar graphs represent averages for the group. There was a statistically significant increase in the concentration of milk GLP-1 from fore- to hindmilk (95% CI: (−0.11%, 61.31%), *P* = 0.005).

**Figure 2:**
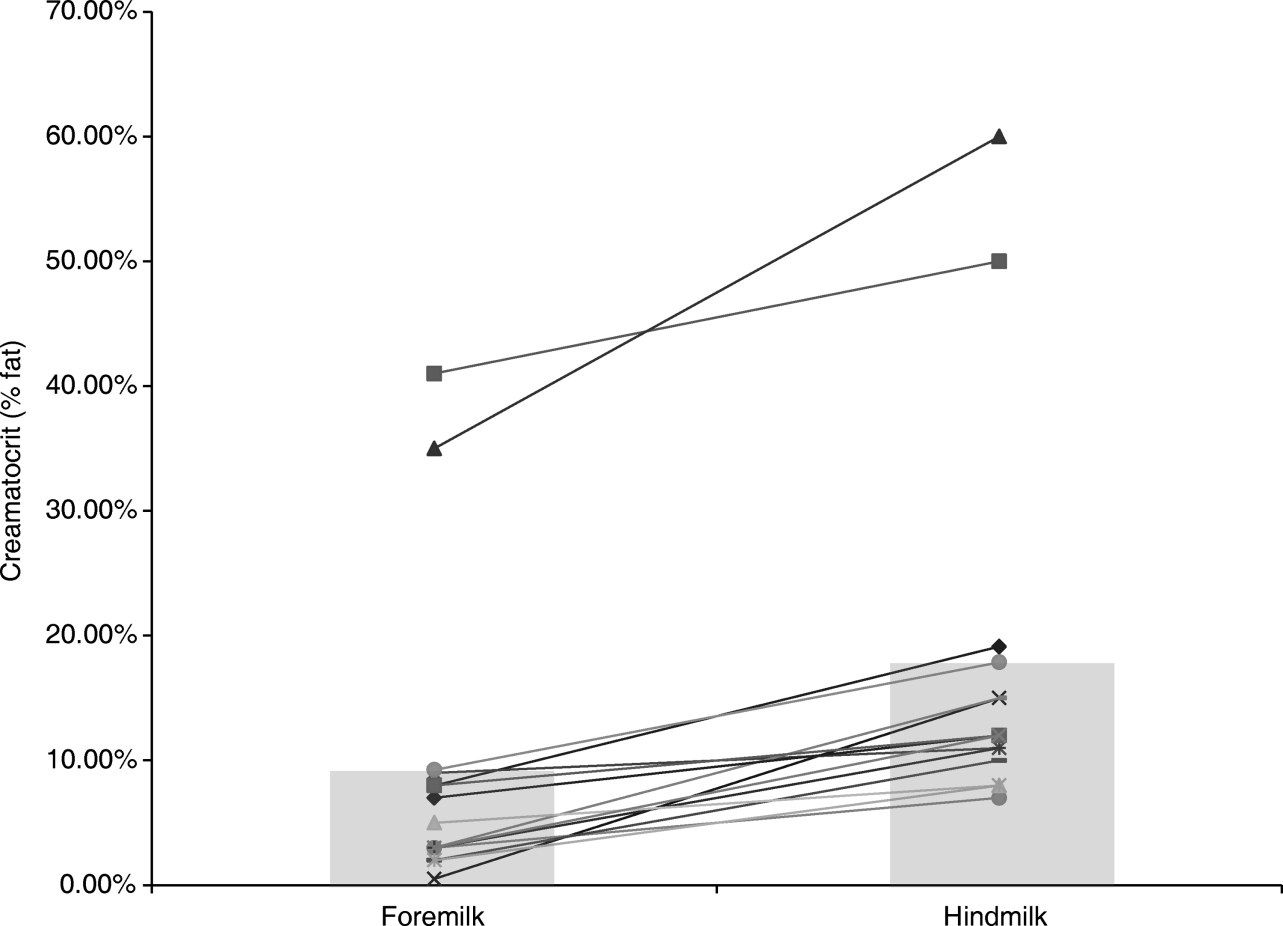
Content of milk fat (creamatocrit) in fore- and hindmilk as measured in 12 postpartum women 1 month after delivery. Each woman is represented by a line in the figure and bar graphs represent averages for the group. There was a statistically significant increase in the fat content of milk from fore- to hindmilk (95% CI: (4.70%, 12.80%), *P* = 0.001).

GLP-1 concentration increased 30.6% (*P* = 0.051, 95% CI: −0.1-61.3%) from fore- to hindmilk (Figure 1, Table3). Fat content also increased 8.8% by volume (*P* = 0.001, 95% CI: 4.7-12.8%) in hindmilk compared to foremilk (Figure 2, Table3). Milk concentrations of leptin (*P* = 0.205) and PYY (*P* = 0.909) did not change from fore- and hindmilk (Table3). Because concentrations of milk leptin did not change between fore- and hindmilk, the average concentration of fore- and hindmilk samples was used in all subsequent analyses.

**Table 3:** Change in milk hormone concentration and fat content between fore- and hindmilk

	Mean difference (%)	*T*	*P*	95% CI
Change in milk PYY (% increase)	−0.52	−0.117	0.909	(−10.21%, 9.17%)
Change in milk GLP-1 (% increase)	30.60	2.171	0.051	(−0.11%, 61.31%)
Change in milk leptin (% increase)	13.33	1.342	0.205	(−8.32%, 34.97%)
Change in milk fat content (%) (*n* = 12)	8.75	4.759	0.001	(4.70%, 12.80%)

GLP-1, glucagon-like peptide 1; PYY, peptide YY. Change was calculated as [(hind-fore)/fore] for PYY, GLP-1, and leptin, and was the difference in means for fat content.

### Correlations between milk hormone concentrations and maternal adiposity

**Figure 3:**
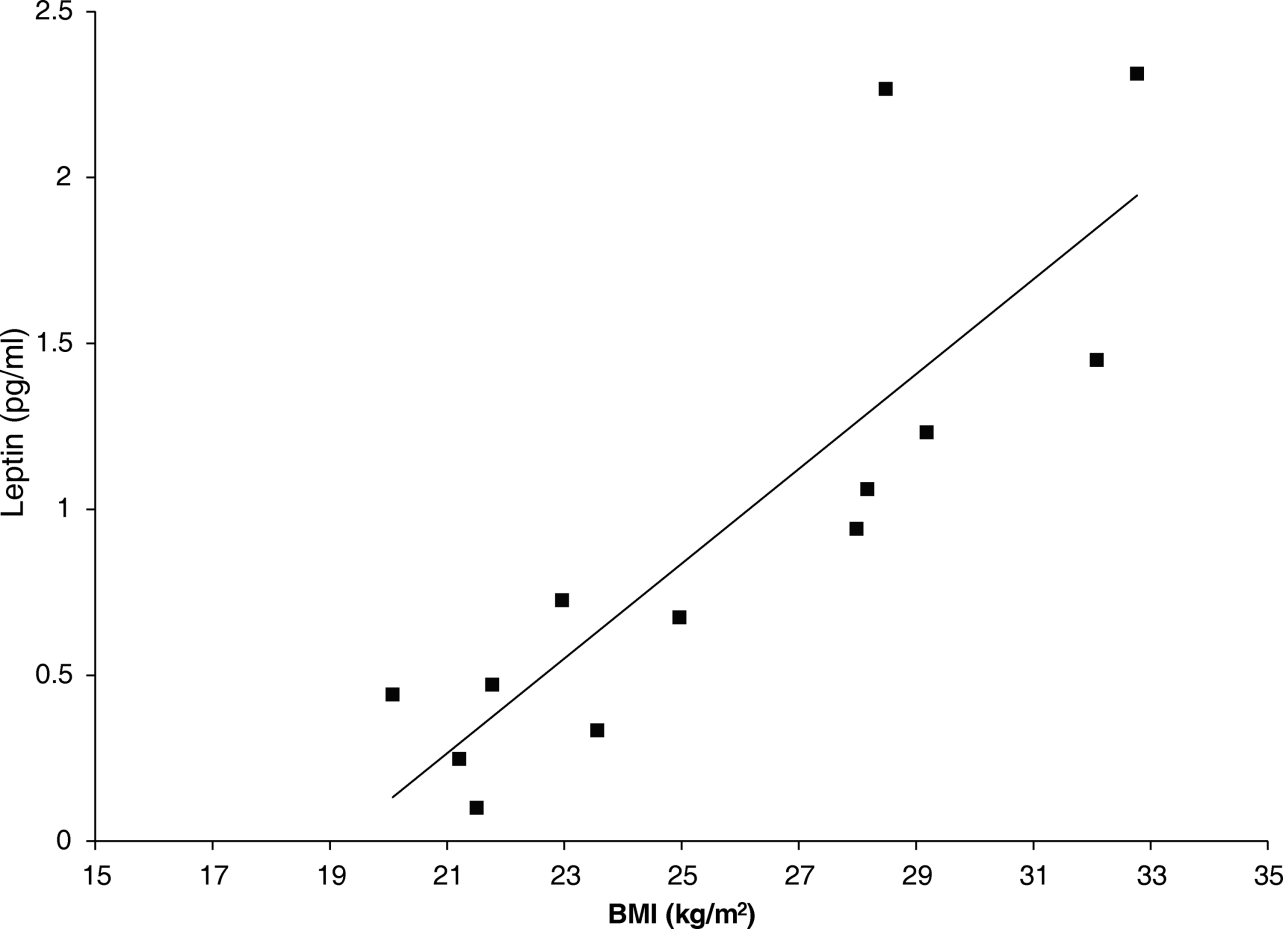
Concentration of leptin in averaged milk compared to maternal BMI measured in 13 postpartum women 1 month after delivery. There was a significant positive relationship between averaged milk leptin and maternal BMI (*r* = 0.85, *P* < 0.001).

Concentration of average milk leptin was correlated to maternal body weight (*r* = 0.79, *P* = 0.001), BMI (*r* = 0.82, *P* = 0.001), fat mass (*r* = 0.86, *P* < 0.001), and percentage body fat (*r* = 0.81, *P* = 0.001) (Figure 3). Foremilk GLP-1 was correlated with maternal body weight (*r* = 0.55, *P* = 0.051). No other correlations were observed.

### Correlations between milk fat content and maternal adiposity

The fat contents of both fore- and hindmilk were correlated with maternal fat mass (*r* = 0.65, *P* = 0.022; *r* = 0.67, *P* = 0.018), BMI (*r* = 0.65, *P* = 0.024; *r* = 0.75, *P* = 0.005), body weight (*r* = 0.63, *P* = 0.028; *r* = 0.59, *P* = 0.044), and percentage body fat (*r* = 0.55, *P* = 0.064; *r* = 0.63, *P* = 0.029) (*n* = 12 for all milk fat correlations). These correlations did not change after adjustment for pre-pregnancy weight or weight gain during pregnancy. Additionally, the change (fore- to hindmilk) in the percent fat of milk samples was strongly related to BMI (*r* = 0.68, *P* = 0.014) and tended to correlate with percentage body fat (*r* = 0.56, *P* = 0.061). No other correlations were observed.

### Relations between milk fat and hormone concentrations and infant anthropometrics

**Figure 4:**
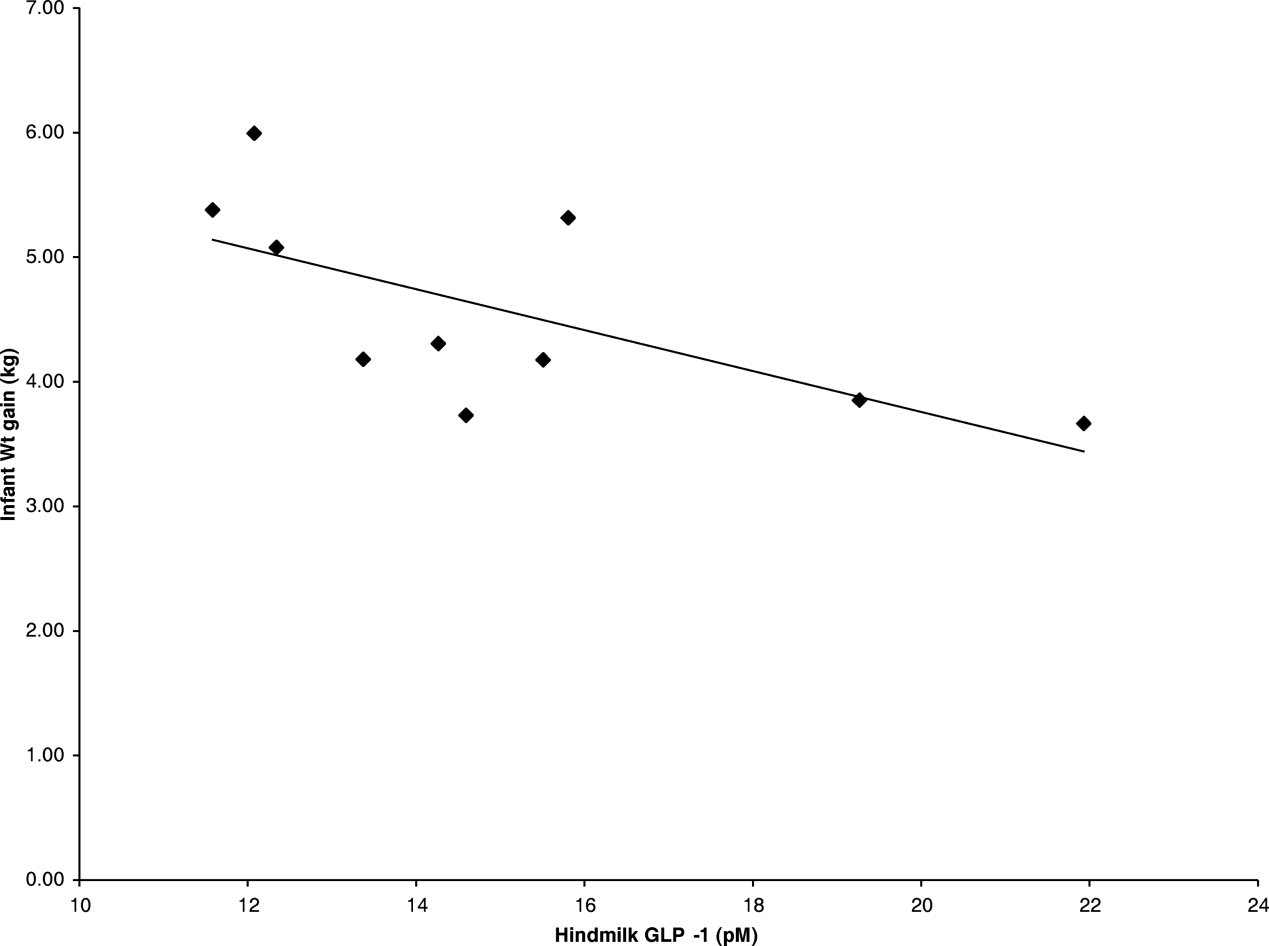
Concentration of hindmilk GLP-1 measured at one month postpartum compared to infant weight gain from birth to six months of age. There was a significant negative correlation between hindmilk GLP-1 and infant weight gain (*r* = −0.67, *P* = 0.034).

Associations between milk GLP-1, PYY, and leptin concentrations or fat content and infant anthropometrics were not observed at 1 month after delivery. Only the percent increase in milk fat content was correlated to infant weight gain at 1 month (*r* = 0.67, *P* = 0.024). However, the concentration of hindmilk GLP-1 at 1 month after delivery was negatively correlated with infant weight gain over the first 6 months (*r* = −0.67, *P* = 0.034; *n* = 10) (Figure 4**)**. There was a trend for this relationship (*r* = −0.69, *P* = 0.057; *n* = 10) at 12 months after birth. Additionally, the concentration of hindmilk GLP-1 was negatively correlated to the infant weight-for-length percentile at 6 months (*r* = −0.64, *P* = 0.046; *n* = 10). No other correlations were observed.

## Discussion

In the current study, all three appetite hormones (GLP-1, PYY, and leptin) were detected in breast milk. Although results for leptin and PYY confirm previous reports, this is, to our knowledge, the first report that GLP-1 is present in human milk. These results also confirm that PYY is present in both fore- and hindmilk. Concentrations of PYY and leptin detected in the current study are similar to those previously reported. The average milk PYY concentration was 39 pg/mL, which is slightly higher than the 23 pg/mL reported by Berseth et al. 15 in breast milk also obtained 4 weeks postpartum. In contrast to the single published study demonstrating the presence of PYY in milk, a whole body of literature exists regarding the presence, concentration, effects, and determinants of leptin in breast milk. Theaverage concentration of milk leptin in the current study, 1.0 ng/mL, falls within the range reported previously, varying from 0.2 to 8.0 ng/mL 11–23. The variability in reported milk leptin concentration could be because of procedural differences in milk processing, including skimming and sonification. Skimming reportedly decreases leptin concentration whereas sonification increases detection 24.

GLP-1, PYY, and leptin are key anorexigenic hormones that promote satiation and decrease the desire to eat. When infused intravenously, all three hormones decrease energy consumption at subsequent meals 18,19. GLP-1 and PYY are produced in intestinal L-cells 16–17, and released in response to nutrient ingestion 16–17. As plasma concentrations of these hormones increase in adults within 10-30 minutes of nutrient ingestion 16–26, they are referred to as short-term appetite hormones. These peptide hormones bind to receptors in the arcuate nucleus of the hypothalamus and inhibit neuropeptide Y-expressing neurons 27,28. These peptides may also act as neuromodulators, promoting satiation at the brainstem via vagal nerve afferents 17,28. In adults, leptin is produced mainly in adipocytes and to a small extent in the gastric mucosa 9. Leptin circulates in plasma at concentrations relative to body adiposity 31 and may communicate the status of body energy reserves to the arcuate nucleus 9. In the arcuate nucleus, leptin binds its receptor, inhibiting neural secretion of neuropeptide Y, a potent stimulator of hunger 32. Lacking an acute response to meal ingestion, leptin is typically considered a long-term appetite regulator.

In order for appetite hormones in milk to have physiological effects in the infant, the hormones must be able to survive gastric digestion and bind a receptor or be endocytosed. Although there is no direct evidence of these processes, a study in human infants found correlations between milk leptin concentration and subsequent plasma leptin concentration in the infants 60 minutes after nursing 33, suggesting milk hormones are biologically active in the infant. Additional evidence is provided by a study by Casabiell et al. 10, which suggests labeled leptin injected into rat mothers is transferred to the milk and can be detected in the circulation of nursing rat pups.

If the appetite hormones are biologically active in the infant, the observed increase in the milk GLP-1 concentration across the feeding may serve as a satiety signal to terminate infant feeding, given that GLP-1 decreases food intake and enhances satiety 19. The initial low concentration of GLP-1 in foremilk would presumably allow relatively unrestricted milk consumption at the start of nursing, followed by an increased concentration in hindmilk, which could potentially signal satiety and contribute to the cessation of feeding. This change in breast milk composition is mirrored by the increase in fat content commonly observed between fore- and hindmilk 11–34. In agreement, fat content of milk in the current study increased, on average, almost 9% by volume, from fore- to hindmilk. The changing composition of breast milk during a single feeding is unique to breast milk given from the breast, as opposed to a bottle. In a recent study by DiSantis et al. 35, infants fed breast milk from a bottle (mixed milk) had lower satiety responsiveness at 3-6 years of age compared to infants who received breast milk from the breast, suggesting the dynamic nature of breast milk may be an additional factor impacting appetite regulation. However, Bartok 36 did not observe any clinically significant differences between these two groups.

In contrast to GLP-1, the concentration of PYY did not change from fore- to hindmilk. Because milk concentrations of PYY were static, there may be important yet unrecognized biological differences between GLP-1 and PYY and/or differences in their synthesis or secretion by mammary tissue. The change in GLP-1 concentration over the relatively short period of time between fore- and hindmilk suggests it is most likely of breast-tissue origin. However, future studies addressing whether the mammary gland is capable of synthesizing this peptide are needed. As for PYY, the concentration of plasma PYY was roughly four times that found in breast milk, as reported by Berseth and colleagues 15 is more suggestive of diffusion from blood than synthesis in mammary tissue.

Supportive of the study hypothesis, the concentration of milk leptin was similar between fore- and hindmilk, providing further evidence that leptin is stable across a single feeding and may not be important for short-term appetite regulation. Similar to leptin's role in adults, concentrations of milk leptin may play a role in long-term energy balance and regulation of infant growth. Milk leptin concentrations are inversely related to infant growth and BMI between 6 and 24 months of age 37–38. Animal studies suggest that oral leptin exposure during the neonatal period may be necessary for development of neural connections important for appetite regulation 32. Rats treated for 20 days after birth with oral leptin had decreased body mass and adiposity at 6 months 39. It has been suggested that breast milk leptin may be involved in metabolic programming, influencing weight regulation during infancy and later life 8.

As expected, concentrations of leptin in both fore- and hindmilk were related to multiple markers of maternal adiposity. The correlation between maternal BMI and milk leptin concentration has been previously reported 14,38, but is not a universal finding 33. Although noted previously in mixed milk, the current study is first to observe the correlation with maternal adiposity in both fore- and hindmilk. Correlations have also been reported between maternal plasma and milk leptin concentrations 33. Milk leptin concentration correlates negatively with infant growth in the first two years of life 37–38, suggesting that breastfeeding may confer additional obesity-protective effects for infants of overweight mothers.

In the current study, maternal BMI was also correlated with the milk fat content of both fore- and hindmilk. This finding could be a result of the higher plasma triglyceride concentrations commonly found in individuals with higher BMI, or higher concentrations of hormones correlated to body adiposity, the most likely candidate being leptin.

In addition to leptin and milk fat, maternal adiposity is also correlated with GLP-1 in foremilk, but not hindmilk. A possible explanation for this finding is that leptin increases secretion of GLP-1, but not PYY 9. The lack of correlation between maternal adiposity and GLP-1 concentration in hindmilk is intriguing and may be because of other factors controlling hormone concentrations, including release of stored hormones from mammary tissue. A provocative result of this study was the correlation between hindmilk GLP-1 and infant growth from birth to 6 months of age, which suggests that the appetite-suppressing hormone may have an impact on growth regulation of the infant. As GLP-1 was also found in the current study to increase over the course of the feeding, this hormone may have an impact on growth regulation through regulation of individual feedings. The lack of a significant correlation between hindmilk GLP-1 collected 1 month postpartum and infant growth from birth to 12 months may be a result of the introduction of solid foods around 6 months after birth, which decreases the infant's intake of breast milk and introduces a variety of dietary factors that could affect weight gain.

Although this pilot study provides intriguing results suggesting appetite hormones in breast milk may influence infant feeding, the study was limited by sample size, RIA sensitivity, use of physician-obtained infant anthropometrics in a limited number of cases, and a focus only on satiety hormones. Certainly additional research is needed to confirm the current findings and further explore how synthesis and/or secretion of milk appetite hormones are regulated and determine the origin of these hormones. Although this study evaluated changes in the hormone and fat content of milk at 4-5 weeks postpartum, the importance of appetite regulation and metabolic programming may be greater earlier on in the neonatal period as suggested by animal studies 32, when the infant's appetite regulation system is maturing.

In conclusion, this pilot study has identified a potentially bioactive component in breast milk that changes across a single feeding. The presence of this hormone and its changing concentration suggests that it might regulate infant appetite during individual feedings. Additionally, differences in appetite hormone exposure may create permanent changes in the way the brain reacts to appetite hormones and satiety cues 8, which may help control appetite in breastfed infants. Lastly, the finding that concentrations of milk GLP-1 change from fore- to hindmilk (along with milk fat) suggests that delivering human milk from the breast may differ from the provision of mixed-milk that results from pumping and feeding by bottle.
